# Butyrate regulates inflammatory cytokine expression without affecting oxidative respiration in primary astrocytes from spontaneously hypertensive rats

**DOI:** 10.14814/phy2.13732

**Published:** 2018-07-24

**Authors:** Tao Yang, Vermali Rodriguez, Wendi L. Malphurs, Jordan T. Schmidt, Niousha Ahmari, Colin Sumners, Christopher J. Martyniuk, Jasenka Zubcevic

**Affiliations:** ^1^ Department of Physiological Sciences College of Veterinary Medicine University of Florida Gainesville Florida; ^2^ Department of Physiology and Functional Genomics College of Medicine University of Florida Gainesville Florida; ^3^ Department of Physiological Sciences and Center for Environmental and Human Toxicology University of Florida Genetics Institute Interdisciplinary Program in Biomedical Sciences Neuroscience College of Veterinary Medicine University of Florida Gainesville Florida

**Keywords:** Blood pressure, butyrate, glial cells, microbial metabolites, neurogenic hypertension

## Abstract

Neurons and glia exhibit metabolic imbalances in hypertensive animal models, and loss of metabolic homeostasis can lead to neuroinflammation and oxidative stress. The objective of this study was to determine the effects of the microbial metabolite butyrate on mitochondrial bioenergetics and inflammatory markers in mixed brainstem and hypothalamic primary cultures of astrocytes between normotensive (Sprague‐Dawley, S‐D) and spontaneously hypertensive (SHR) rats. Bioenergetics of mitochondria in astrocytes from normotensive S‐D rats were modified with butyrate, but this was not the case in astrocytes derived from SHR, suggesting aberrant mitochondrial function. Transcripts related to oxidative stress, butyrate transporters, butyrate metabolism, and neuroinflammation were quantified in astrocyte cultures treated with butyrate at 0, 200, 600, and 1000 *μ*mol/L. Butyrate decreased catalase and monocarboxylate transporter 1 mRNA in astrocytes of S‐D rats but not in the SHR. Moreover, while butyrate did not directly regulate the expression of 3‐hydroxybutyrate dehydrogenase 1 and 2 in astrocytes of either strain, the expression levels for these transcripts in untreated cultures were lower in the SHR compared to S‐D. We observed higher levels of specific inflammatory cytokines in astrocytes of SHR, and treatment with butyrate decreased expression of Ccl2 and Tlr4 in SHR astrocytes only. Conversely, butyrate treatment increased expression of tumor necrosis factor in astrocytes from SHR but not from the S‐D rats. This study improves our understanding of the role of microbial metabolites in regulating astrocyte function, and provides support that butyrate differentially regulates both the bioenergetics and transcripts related to neuroinflammation in astrocytes from SHR versus S‐D rats.

## Introduction

A number of physiological and molecular mechanisms are proposed to underlie the onset and progression of neurogenic hypertension (HTN). These include, but are not limited to, factors regulating sympathetic outflow and sympathoadrenal system (Mann 2003; Houston 2011), decoupling of respiratory and sympathetic activities (Zoccal and Machado 2011; Fisher and Paton 2012; Zubcevic et al. 2014b), vascular inflammation in the central nervous system (CNS) (Waki et al. 2011; Zubcevic et al. 2011), and glial activation underlying neuroinflammatory responses in various cardioregulatory regions (Santisteban et al. 2015; Shen et al. 2015; Bhat et al. 2017). In addition to these examples, mitochondrial dysregulation and oxidative stress are proposed to be major factors in pathophysiology of neurogenic HTN (Peterson et al. 2006; Zimmerman and Zucker 2009; Li et al. 2013; de Queiroz et al. 2015; Chan and Chan 2017).

There is also strong evidence that gut dysbiosis is associated with an array of human diseases, and a growing number of studies support an association between neurogenic HTN and the gut microbiota (Yang et al. 2015; Galla et al. 2017; Santisteban et al. 2017; Zubcevic et al. 2017). In addition, recent studies by Pluznick et al. (2013) suggest a role for short chain fatty acid (SCFA) signaling in the regulation of blood pressure. Butyrate, for example, is a major metabolite produced in the colonic lumen by bacterial fermentation of dietary fiber, which is proposed to have systemic and CNS benefits by acting as an anti‐inflammatory agent in some cases (Canani et al. 2011; Brahe et al. 2013; Ohira et al. 2013; Singh et al. 2014; Bourassa et al. 2016). Although butyrate has been shown to regulate the colonic metabolic process and inflammatory response (Pluznick et al. 2013), its direct role in neuroinflammatory and metabolic responses in the brain has not yet been studied. This is highly relevant for neurogenic HTN as butyrate is able to cross the blood brain barrier (Braniste et al. 2014), and studies suggest that the reduction in butyrate‐producing bacteria is associated with HTN (Yang et al. 2015). Indeed, new evidence demonstrates that butyrate can improve metabolic performance in states of specific neurological disorders (Joseph et al. 2017). Thus, there is merit in investigating the therapeutic role of SCFAs such as butyrate in other diseases presenting with CNS dysfunction such as HTN.

The objective of this study was to directly measure the effects of butyrate on mitochondrial bioenergetics of mixed brainstem/hypothalamic primary astrocytes cultured from both normotensive (Sprague‐Dawley, S‐D) and spontaneously hypertensive rats (SHR). The SHR, as a rodent model of neurogenic HTN, is characterized by dysfunctional autonomic nervous system (ANS), overactive renin‐angiotensin system (RAS), activated peripheral and central immune system (IS), and mitochondrial dysfunction in the brain (Zimmerman and Zucker 2009; Zubcevic et al. 2014a; Santisteban et al. 2015). Moreover, to elucidate potential molecular mechanisms associated with regulation of mitochondrial function, several transcripts related to oxidative stress, butyrate transport, signaling and metabolism, and neuroinflammation were measured in primary cultures of astrocytes generated from both rat strains following butyrate treatment. Noteworthy is that several studies demonstrate a close association between impaired mitochondrial bioenergetics and neuroinflammation in astrocytes under different disease conditions (Trudler et al. 2015; Wang et al. 2017; Sarkar et al. 2018). Thus, we hypothesized that impaired mitochondrial function will be associated with increased expression of inflammatory cytokines in astrocytes of SHR. Moreover, considering the lower levels of butyrate‐producing bacteria in the gut of SHR (Yang et al. 2015), we propose that treatment with butyrate will improve both the inflammatory and bioenergetics profiles in the SHR astrocytes.

## Methods

### Culturing of astrocytes

Astrocytes were prepared and cultured as previously described with slight modification (Wang et al. 1989; Sumners et al. 1991) (UF IACUC#201708217). Briefly, brainstem and hypothalami of 2 days old male and female S‐D and SHR pups were pooled separately (~9 pups per culture), and primary astrocytes (passage 1) were cultured in DMEM, supplemented with 10% FBS and 1% pen/strep, for 14 days, prior to treatment with gradient concentrations (i.e. 0.2 mmol/L, 0.6 mmol/L, and 1 mmol/L) of butyrate for 5 h. Cultured in this way, our astrocytes are >95% pure, as per our published protocols (Kopnisky et al. 1997). One limitation to using primary astrocytes pooled from both sexes and both hypothalamus and brainstem is that the method does not take into account the heterogeneity of astrocyte both in sex and neuroanatomical location. However, we point out that our culturing approach from neonates is comparable to that of other investigators in the field who investigate in vitro mechanisms in cells of cardioregulatory regions (Sun et al. 2004). Attributable to the size of the newborn pups, it is technically challenging to isolate specific brain regions. This is why brainstem and hypothalami, containing major cardioregulatory regions, are pooled. Moreover, it is not possible to sex the pups until they are close to weaning or have been weaned.

We examined the response of astrocytes at 5 h post‐treatment. This time point was chosen as an intermediate for capturing gene expression changes as well as mitochondrial bioenergetics perturbations; however, it is acknowledged that earlier (or later) time points may yield different expression profiles than that observed here. The experiment was performed on two separate occasions to yield sufficient cultures for gene expression and mitochondrial experiments. The S‐D strain was chosen as the normotensive control to SHR as has been done previously (Coyle 1986; Kingsley and Snyder 1988; Azar et al. 2012), as opposed to Wistar‐Kyoto rats, which have been linked with neurochemical and behavioral traits that may be confounding to our study (De La Garza and Mahoney 2004; McAuley et al. 2009; Burke et al. 2016), especially considering the role of microbiota metabolites in anxiety and depression (Neufeld et al. 2011; Chen et al. 2018).

**Figure 1:**
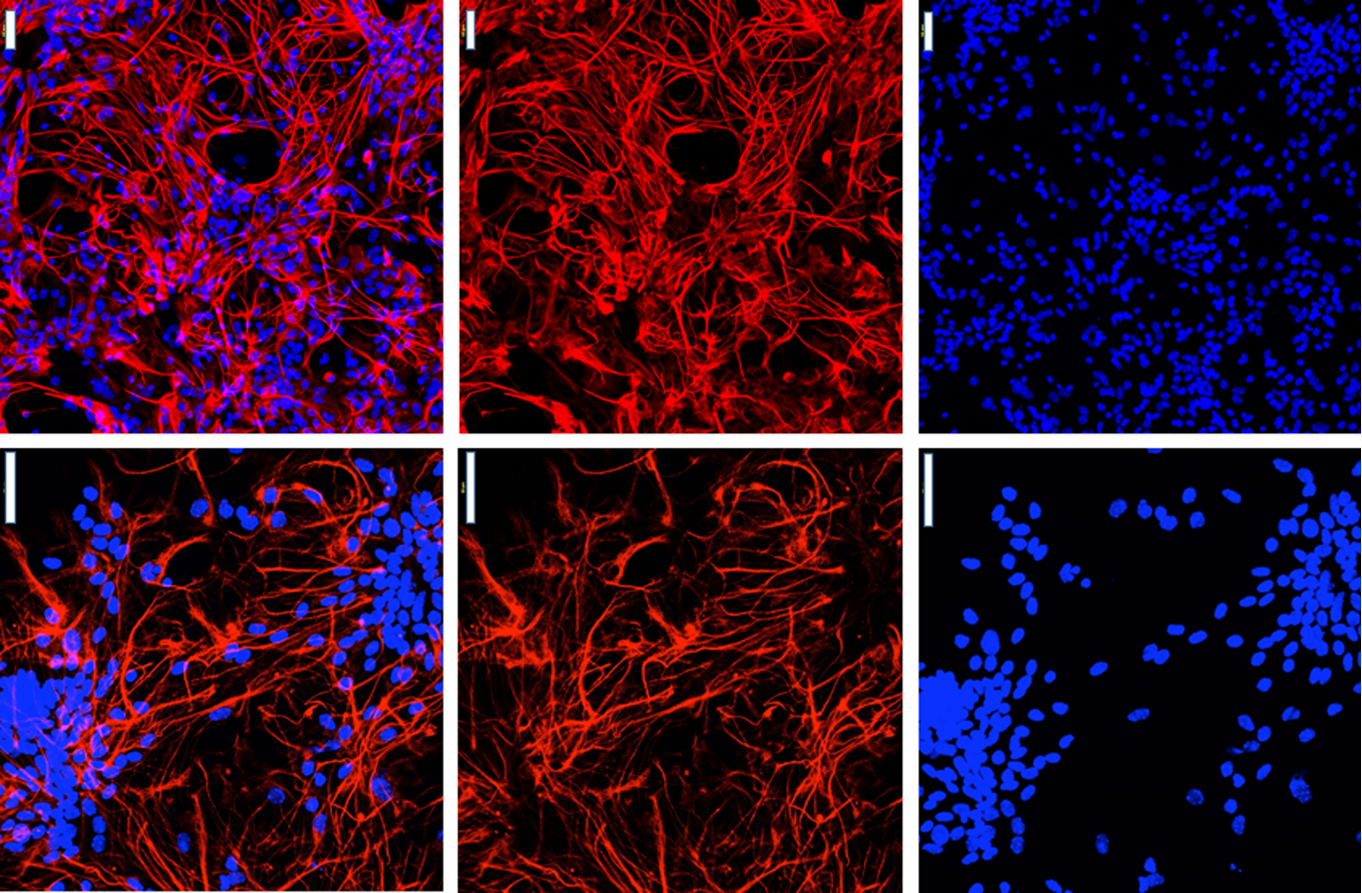
Morphology and verification of primary astrocytes cultured from mixed brainstem and hypothalamic brain regions. Cultured astrocytes stained with GFAP and DAPI (Left panel merge) were visualized by immunofluorescence with goat anti‐mouse Alexa Fluor^®^ 594 (200× and 400×) (*n* = 3).

Briefly, newborn pups were anesthetized with CO_2_. Hypothalami and brainstem were removed and dissected into pieces. Microdissected pieces were incubated in the 2.5 mg/mL trypsin solution at 37°C for 5 min. An additional 5 min incubation was performed in the presence of DNase I at concentration of 0.16 mg/mL. Following this, 20 mL of DMEM (10% FBS, 1% pen/strep) was added to the samples and they were centrifuged for 5 min to spin down all of the cells and its connective tissue. The final pellet was then resuspended with an additional amount of DMEM (10% FBS, 1% pen/strep) and filtered in the suspension. Pellets were then rinsed twice with DMEM and were placed at the same confluency in dishes that were pretreated with 0.01 mg/mL poly I lysine solution. Purity of cells was confirmed by immunohistochemistry for glial fibrillary acidic protein (GFAP) (Fig. 1). Briefly, cells were incubated with 2% PFA for 20 min followed by 3X wash with PBS. This was followed by 0.05% Triton X 100 for 30 min at room temperature (3X PBS wash), followed by blocking with 10% normal serum for 1 h at room temperature. Primary antibody (1:600) was incubated with the cells overnight at 4°C (3X PBS wash), followed by the secondary antibody (1:1000) at 45 min at room temperature (3X PBS wash). For the mount with DAPI, the primary antibody used was anti‐GFP Santa Cruz sc‐33673 lot #J1717 and the secondary antibody goat anti‐MS 594 flouromount G for mounting. After a final wash in PBS, the cells were mounted using Fluoromount‐G with DAPI and imaged on a Nikon confocal microscope with a 20× and 40× objective.

### Mitochondrial bioenergetics

Flasks containing confluent astrocytes at 70–80% were collected using 0.25% trypsin‐ethylenediaminetetraacetic acid (EDTA) (2 mL; Gibco) for mitochondrial bioenergetics assays after being treated with 0.6 mmol/L butyrate for 5 h. Astrocytes were pelleted by centrifugation (100 g for 6 min at 4°C) and then gently washed (3x) in ice‐cold 1X phosphate‐buffered saline (PBS) prior to conducting the mitochondrial bioenergetics assay. Mixed brainstem/hypothalamic astrocytes were seeded at 5.0 × 10^4^ cells per well in a Seahorse V7 cell culture plate overnight in seeding medium (1:10 RPMI 1640, 1% FBS, 10 U/mL penicillin, 10 *μ*g/mL streptomycin, 25 ng/mL amphotericin B) (*n* = 3 replicates/group). Cells were distributed equally across all wells and allowed to equilibrate for 24 h in incubation media. The same number of mixed astrocytes were used for each experimental plate. Cells were washed with incubation media (bicarbonate‐free RPMI 1640, 2 mmol/L Ala‐Gln, and 1 mmol/L pyruvate), treated, and placed into the Seahorse XFe24 Extracellular Flux Analyzer (Agilent). Background wells in the plate numbered *n* = 4. A bioenergetics profile was generated using specific uncoupling agents and mitochondrial toxicants. Each measurement cycle was as follows: 2:00 min. mix, 1:00 min. wait, and 3:00 min. measure. There were three cycles for basal oxygen consumption rate. Each mitochondrial toxicant was then injected in sequential order (final concentration of 0.5 *μ*mol/L oligomycin, 3 *μ*mol/L carbonyl cyanide‐4 (trifluoromethoxy)phenylhydrazone (FCCP), and 0.3 *μ*mol/L antimycin. Experiments were conducted separately for astrocytes harvested from S‐D and SHR rats. All statistical analyses were performed using ANOVA in GraphPad Prism 6.0 (Graphpad Software, Inc., La Jolla, CA). Comparisons were conducted within an experimental plate, relative to the control group. To be cautious, direct comparisons of OCR across each experimental runs was not conducted.

### Real‐time PCR

For qPCR, cultures were treated with one dose of either 0, 0.2, 0.6, or 1 mmol/L butyrate. The butyrate concentrations were determined based on previous publications indicating 0.4–0.7 *μ*mol/g butyrate per wet brain (≈mmol/L) (Liu et al. 2015). Thus, the concentrations of butyrate used in the present study were within the range from the low end to the potential transient high peak of reported butyrate levels in the whole rat brain. Following the treatments, 2–3 × 10^6^ cells were collected and lysed in 1 mL of Trizol. Total RNA was isolated according to the manufacture's protocol. The ratio of OD_260_/OD_280_ and the concentration of total RNA were determined by sampling 1.2 *μ*L of extracts on NanoDrop (Thermo Fisher Scientific, Waltham, MA). A total of 500 ng of RNA from each samples was used to synthesize cDNA by using High Capacity cDNA Reverse Transcription Kit (#15596026, Thermo Fisher Scientific, Waltham, MA). Inflammation‐related genes and butyrate sensing receptors were tested. Primers are reported in Table 1. Real‐time PCR was performed on CFX96 Touch™ Real‐Time PCR Detection System (Bio‐Rad, Hercules, CA, USA). The PCR conditions were as follows: 95°C for 3 min, followed by 40 cycles of 95°C for 30 sec, primer annealing at 60°C for 30 sec, and 72°C for 30 sec. Dissociation curves were generated, starting at 65°C and ending at 95°C with increments of 0.5°C every 5 sec. Gene expression was normalized to GAPDH and was determined using the relative ΔCt method based on the method described (Pfaffl 2001). No reverse transcriptase control was also included in the reaction to ensure no DNA contamination.

**Table 1:** List of primer sequences (5′ to 3′) used to measure transcripts in the study

Name	Forward primer	Reverse primer
Sod1	AATGTGTCCATTGAACATCGTGTGA	GCTTCCAGCATTTCCAGTCTTTGTA
Sod2	AGGGCCTGTCCCATGATGTC	AGAAACCCGTTTGCCTCTACTGAA
Cat	CCCAGAAGCCTAAGAATGCAA	TCCCTTGGCAGCTATGTGAGA
Gpx	GCTGTGCGCGCTCCAT	ACCATGTGCCCATCGATGT
Bdh1	GAGGGTCTTGAGAAACAGAGGC	GGTGGCTCCCACAACGAG
Bdh2	GGATTGCACTGCAGGATCCAC	TCCGTGGTGGACAAAACCAG
Mct1	AAGCGGAGGAAAAGAAGAGG	TAGACTAGGGGCCAGCAGAA
Atct1	GGGTGCAGGTCTACCCATTG	GGTGTTGCTCCTCTGCTCAT
Hmgcs1	ATCGCGTTTGGTGCCTGAAG	AAGGGCAACGATTCCCACAT
Oxct1	CACCTTGCTACCCACTCCTG	CACAACCCGAAACCACCAAC
Oxct2a	CACAACCCGAAACCACCAAC	CGCGGATCATGGCAAAAGAG

Ccl2	Rn00580555_m1, Thermo Fisher
Il1b	Rn00580432_m1, Thermo Fisher
Olr59	Rn01508979_m1, Thmero Fisher
Tlr4	Rn00569848_m1, Thermo Fisher
Agtr1a	Rn02758772_s1, Thermo Fisher
Tnf	Rn01525859_g1, Thermo Fisher

### Sprague‐Dawley and SHR‐dependent effects of butyrate in astrocytes

A gene interaction network for expression targets was built using Pathway Studio v11 (Elsevier) to better visualize similarities and differences in astrocytes of S‐D and SHR following butyrate treatments. Official gene symbols were mapped into Pathway Studio and the expression of the transcript from the highest dose of butyrate relative to the control was used for coloring the networks. Interaction networks were based upon expression, binding, and regulatory interactions and were built using by direct connections with one neighbor. We mapped this interactome using fold change for each transcript investigated in this study data from both SD and SHR. This representation aimed to provide a more global view of the expression network in response to butyrate in the two models.

### Statistics

All statistical analyses were performed using GraphPad Prism 6.0 (Graphpad Software, Inc., La Jolla, CA). For mitochondrial bioenergetics, data were analyzed with a One‐Way ANOVA. For gene expression analysis, data were analyzed by two‐way ANOVA analyses followed by Tukey's multiple comparison to differentiation the significance of butyrate effects on each cell line, and Holm‐Sidak multiple comparison to differentiate the differences between S‐D and SHR with the same butyrate treatment.

## Results

### Mitochondrial bioassays

**Figure 2:**
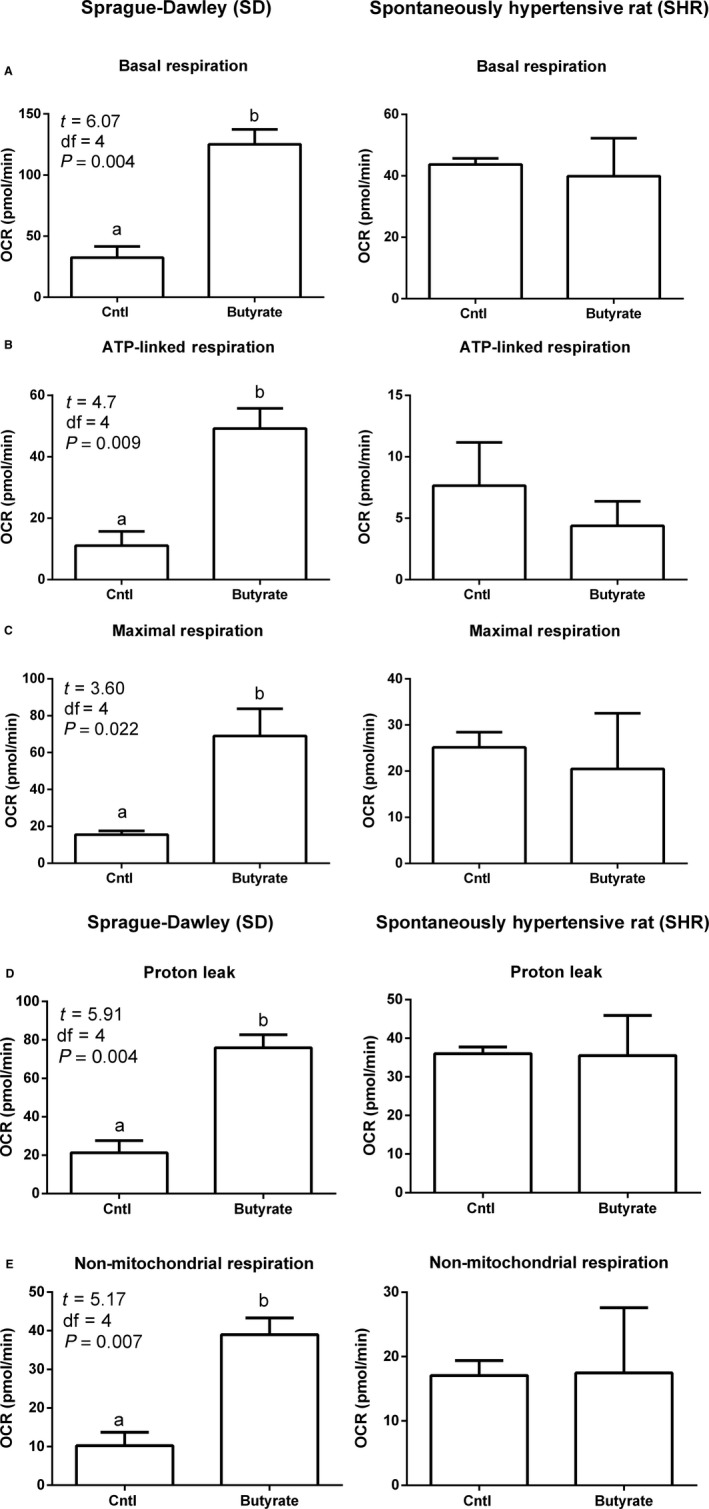
Effect of butyrate on mitochondrial function in mixed brainstem/hypothalamic astrocyte cultures from S‐D and SHR. Incubation of astrocytes with butyrate (0.6 mmol/L for 5 h) increased oxygen consumption rates of astrocytes in S‐D rats but not in the SHR (A–C). Baseline oxygen consumption rate (OCR) in S‐D and SHR astrocytes (A). Oligomycin, FCCP, and antimycin A are used to probe mitochondrial bioenergetics. Each section indicated by the dotted lines is a measured endpoint for mitochondrial function, including basal and maximum respiration. All endpoints calculated from profiles in graph A are presented in graphs B and C (*n* = 3/group); * indicates *P* < 0.05 by two‐way ANOVA followed by Tukey's multiple comparisons test.

In the SD rats, oxygen consumption rates (OCR) were measured in astrocytes following butyrate treatment. The basal respiration of the astrocytes following treatment with butyrate was increased by ~70% (df = 4, *t* = 6.07, *P* = 0.004) (Fig. 2A). Similarly, butyrate significantly increased ATP‐linked respiration (df = 4, *F* = 4.7, *P* = 0.009) (Fig. 2B), maximal respiration (df = 4, *F* = 3.6, *P* = 0.022) (Fig. 2C), proton leak (df = 4, *F* = 5.9, *P* = 0.004) (Fig. 2D), and nonmitochondrial respiration (df = 4, *F* = 5.17, *P* = 0.007) (Fig. 1E). However, in butyrate‐treated astrocytes from SHR, there were no significant differences in any bioenergetics endpoint compared to the SHR astrocytes (Bhat et al. 2017).

### Real‐time PCR

**Figure 3:**
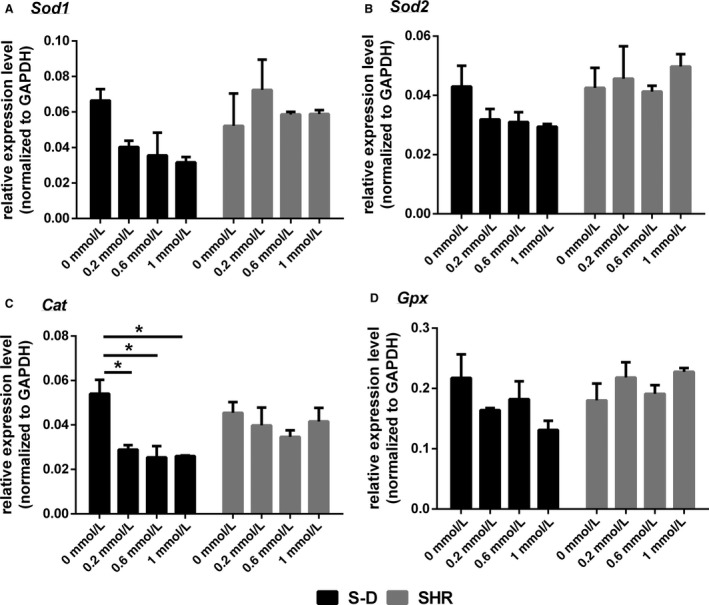
The expression of transcripts related to oxidative damage in mixed brainstem/hypothalamic astrocyte cultures from S‐D and SHR. (A) Sod1, (B) Sod2, (C) Cat, and (D) Gpx. Data are presented as mean ± SE (*n* = 3). Significant differences are indicated among groups, *P* < 0.05 (*), *P* < 0.01 (**), *P* < 0.001 (***).

Three major functional categories of genes were investigated based upon literature evidence for their role in HTN and/or butyrate metabolism. These included transcripts related to oxidative damage, enzymes, and transporters related to butyrate, and transcripts associated with neuroinflammation. For those transcripts related to oxidative stress, there was no change in Sod1 mRNA levels (*F* (3, 8) = 0.89, *P* = 0.49) (Fig. 3A), Sod2 (*F* (3, 8) = 0.46, *P* = 0.72) (Fig. 3B), or Gpx (*F* (3, 8) = 0.25, *P* = 0.86) in astrocytes of either model (Fig. 3D). However, the expression of Cat in the astrocytes was different between control and all three butyrate doses in the SD rats (*F* (3, 8) = 6.00, *P* = 0.020) (Fig. 3C) compared to SHR. This change was not detected in astrocytes collected from SHR following treatment to butyrate.

**Figure 4:**
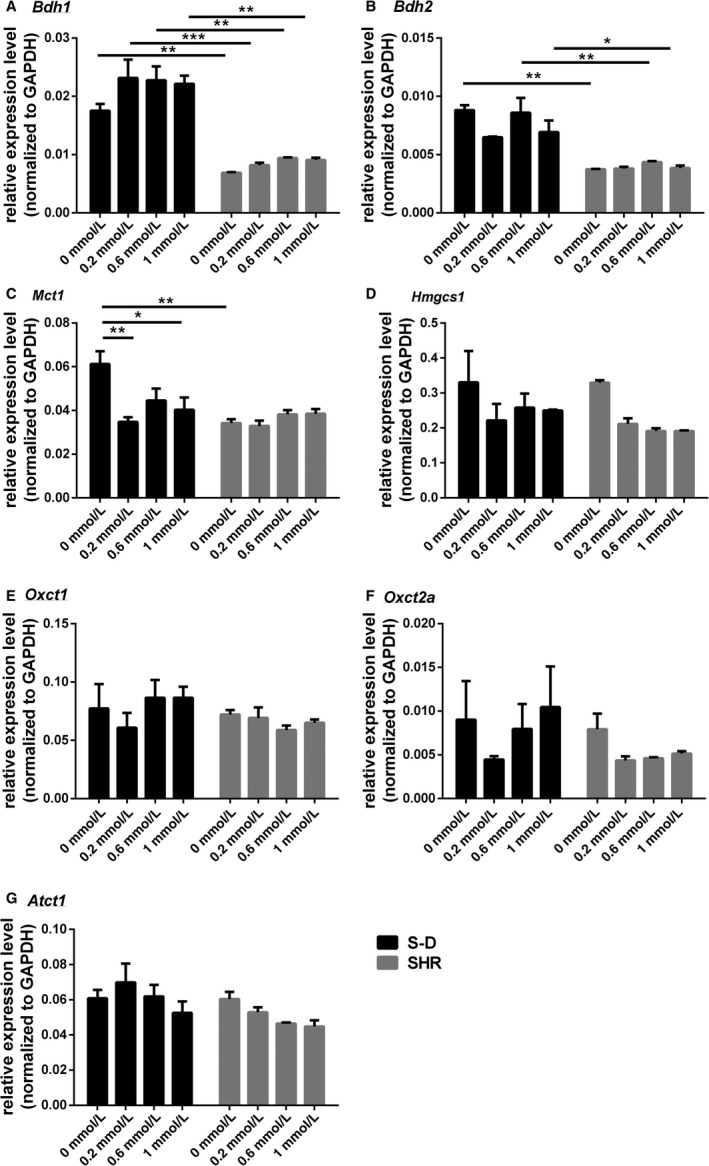
The expression of transcripts related to butyrate transport and metabolism in mixed brainstem/hypothalamic astrocyte cultures collected from S‐D and SHR. (A) Bdh1 (B) Bdh2 (C) Mct1, (D) Hmgcs1, (E) Oxt1, (F) Oxt2a, and (G) Atct1. Data are presented as mean ± SE (*n* = 2–3). Significant differences are indicated among groups, *P* < 0.05 (*), *P* < 0.01 (**), *P* < 0.001 (***).

In terms of transcripts related to butyrate transport and metabolism, there were also marked differences between the astrocytes from S‐D and SHR. For Bdh1, there were differences in the expression of the transcript between the two different rat models (*F* (1, 8) = 141.3, *P* < 0.0001) but no difference in expression following butyrate treatment (*F* (3, 8) = 2.7, *P* = 0.12) (Fig. 4A). This was also the case for Bdh2 expression, and the models differed in expression levels of Bdh2 (*F* (1, 8) = 79.5, *P* < 0.0001) but butyrate did not affect its expression in either model (*F* (3, 8) = 2.4, *P* = 0.15) (Fig. 4B). Mct1, on the other hand differed significantly in expression between S‐D and SHR (*F* (1, 8) = 11.6, *P* = 0.0092) (Fig. 4C), and Mct1 showed differences in expression levels between groups following butyrate treatment (*F* (3, 8) = 4.5, *P* = 0.04) (Fig. 4C). Mct1 expression decreased in steady state abundance with butyrate, but this only occurred in S‐D rats. There was no change in expression levels for Hmgcs1 (*F* (3, 8) = 3.9, *P* = 0.055), Oxct1 (*F* (3, 8) = 0.37, *P* = 0.78), Oxct2a (*F* (3, 8) = 0.99, *P* = 0.45) or Atct1 (*F* (3, 8) = 2.2, *P* = 0.17) in astrocytes of either strain following treatment with butyrate (Fig. 4D–G).

**Figure 5:**
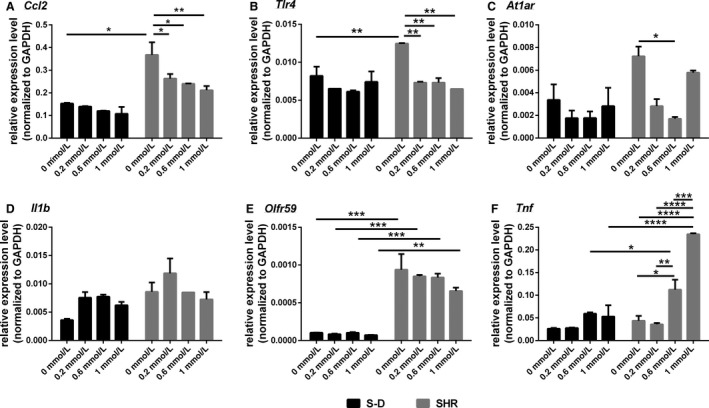
The expression of transcripts related to neuroinflammation in mixed brainstem/hypothalamic astrocyte cultures collected from S‐D and SHR. (A) Ccl2 (B) Tlr4 (C) At1ar, (D) Il1b, (E) Olfr59, and (F) Tnf. Data are presented as mean ± SE (*n* = 3). Significant differences are indicated among groups, *P* < 0.05 (*), *P* < 0.01 (**), *P* < 0.001 (***).

Transcripts related to neuroinflammation were also assessed for regulation by butyrate in S‐D and SHR. Expression levels for CCl2 varied significantly between S‐D and SHR (*F* (1, 8) = 66.7, *P* < 0.0001) and butyrate significantly altered the expression of this transcript in SHR (*F* (3, 8) = 6.4, *P* = 0.016) (Fig. 5A). The same was also true for Tlr4. Butyrate decreased the expression levels of Tlr4 in SHR but not S‐D (*F* (3, 8) = 12.9, *P* = 0.002). This transcript also showed differences in expression between S‐D and SHR (*F* (1, 8) = 7.5, *P* = 0.026) (Fig. 5B). At1ar mRNA levels were also reduced by butyrate in SHR, but not S‐D (*F* (3, 13) = 5.02, *P* = 0.016) (Fig. 5C). Il1b mRNA levels did not differ among groups with butyrate (*F* (3, 8) = 3.21, *P* = 0.083) (Fig. 5D). Olfr59 mRNA levels did not vary with butyrate (*F* (3, 8) = 1.43, *P* = 0.31), but expression levels were different between S‐D and SHR astrocytes (*F* (1, 8) = 176.2, *P* < 0.0001). Astrocytes harvested from SHR showed considerable higher levels of Olfr59 compared to S‐D (Fig. 5E). Lastly, Tnf showed a significant increase in expression in SHR with butyrate (*F* (3, 15) = 29.6, *P* < 0.0001) and levels of the transcript were higher overall in SHR compared to S‐D (*F* (1, 15) = 48.5, *P* < 0.0001) (Fig. 5F).

### Gene interactome

**Figure 6:**
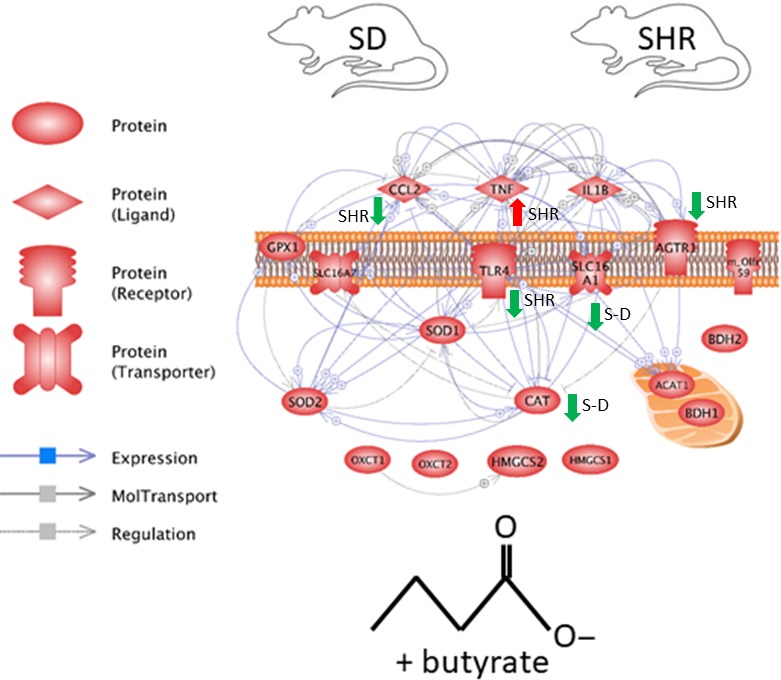
Network summarizing overall gene expression changes in mixed brainstem/hypothalamic astrocyte cultures from S‐D and SHR following butyrate treatment. Green downward pointing arrows depict downregulated, while red upward pointing arrows depict upregulated transcripts following butyrate treatment.

To better illustrate differences in expression patterns for genes measured in this study, we constructed a gene network encompassing all investigated transcripts. The network reveals the interaction of these genes based upon evidence from literature (Fig. 6). Figure 6 summarized our results and illustrates that relative expression of inflammatory transcripts and AT1 receptor was affected by butyrate in the SHR animals, but not S‐D, where all the transcripts that were downregulated following butyrate application are marked by green downward pointing arrow. Conversely, relative expression levels of Cat and butyrate transporter Slc16a1 were downregulated by butyrate treatment in S‐D astrocytes only, as illustrated by green downward pointing arrow.

## Discussion

Neurogenic HTN is present in a significant number of individuals suffering from treatment resistant HTN, in which blood pressure remains high despite the combined use of 3 or more antihypertensive drugs (Acelajado and Calhoun 2010). Neurogenic HTN is a condition that is characterized by exaggerated sympathetic drive, oxidative stress, and neuroinflammation (Wu et al. 2012), among other factors. Moreover, gut dysbiosis has been shown in human and rodent HTN (Yang et al. 2015; Ahmadmehrabi and Tang 2017; Santisteban et al. 2017; Yan et al. 2017), and this revelation has generated exciting avenues for exploring how one may modulate gut microbiota and their end products for novel therapies for HTN and other dysbiosis‐associated diseases.

Butyrate may be one excellent candidate for such novel treatments. Butyrate reportedly exerts many beneficial effects on the gut, immune system, CNS, and the cardiovascular system (Furusawa et al. 2013). In the periphery, it is reportedly anti‐inflammatory (Säemann et al. 2000; Segain et al. 2000). Moreover, due to its ability to cross the blood brain barrier, direct central effects of butyrate are also possible (Stilling et al. 2016). Attributable to this, we sought to investigate its possible role in regulation of function in mixed brainstem/hypothalamic astrocytes in culture and its possible effects on neuroinflammation. Traditionally, astrocytes are considered to be the building blocks of brain microarchitecture, and are involved in regulation of brain homeostasis, synaptic maintenance, and immune defense. However, the role of astrocytes in neuronal‐glial communication in central cardioregulation has recently been revised. More recent studies demonstrated a more active role for astrocytes in regulation of hypothalamic neuronal activity and sympathetic outflow in rodent models of HTN and heart failure (Potapenko et al. 2012, 2013; Naskar and Stern 2014; Kim et al. 2015; Stern et al. 2016). Thus, understanding how astrocytes are regulated by small chain fatty acids such as butyrate could modulate and improve astrocyte function in the context of HTN. As stated in our Methods section, it is important to note that, due to technical challenges, our experiments are performed in mixed brainstem/hypothalamic astrocyte cultures, which are pooled from both sexes. This may present some inherent caveats with regard to interpretation. However, these are standard established methods when investigating effects on cell cultures in cardioregulatory regions (Sun et al. 2004, 2007). Nevertheless, these should be kept in mind when interpreting our results as inherent caveats may exists due to gender and neuroanatomical heterogeneity.

We first examined the effect of butyrate on mitochondrial function in primary mixed astrocyte cultures of both normotensive and hypertensive animals. Butyrate had a positive effect on the oxygen consumption rates of mitochondria in S‐D rats but not SHR, suggesting that the mitochondria may respond aberrantly to butyrate in the SHR. Moreover, while Sod1 and Sod2 expression was not different between S‐D and SHR, nor responsive at the transcript level to butyrate, butyrate treatment suppressed Cat expression in S‐D astrocytes only. In addition, astrocyte expression levels of 3‐hydroxybutyrate dehydrogenase 1 and 2 (Bdh1 and Bdh2) were lower in the SHR; however, we observed no direct effect of butyrate treatment on relative expression of Bdh1 and 2 in either strain. Therefore, astrocyte mitochondrial oxygen consumption and the expression of a key enzyme related to oxidative stress responses, were not modulated by butyrate in the SHR. This may be due to several reasons. Brain mitochondrial dysfunction and gut dysbiosis, characterized by lower levels of butyrate producing bacteria, are documented in rodent models of HTN including the SHR (Zimmerman and Zucker 2009; Yang et al. 2015; Collister et al. 2016; Sun et al. 2017). Noteworthy is that glucose metabolism can also be affected by butyrate in the brain (Ide et al. 1969). Thus, it may be that the observed inappropriate responses of SHR astrocytes to butyrate were due to reduced butyrate availability in this rat strain. Alternatively, the observed responses may be associated with a reportedly prohypertensive environment in the SHR brain. For example, angiotensin II (ANG II), a potent vasoconstrictor involved in pathophysiology of HTN, can modulate glucose metabolism in astrocytes, which can also affect bioenergetics. Data show that ANG II stimulates glucose uptake into cultured astroglia via a pathway that involves AT1 receptors (Tang et al. 1995). In our study, butyrate treatment downregulated the AT1 receptor expression in the SHR but not the S‐D astrocytes, which may account for the lack of a bioenergetics response following butyrate treatment in the SHR. Moreover, the levels of the monocarboxylate transporter member 1 (Mct1) are lower in astrocytes of the SHR compared to S‐D rats. Mct1 is a well‐characterized transmembrane protein that actively transports SCFAs, lactate, pyruvate, and ketone bodies such as acetoacetate and beta‐hydroxybutyrate across the plasma membrane in order to maintain cell homeostasis (Cuff et al. 2005; Thibault et al. 2010), and its downregulation is associated with increased local inflammatory levels (Thibault et al. 2007). Thus, the decline in Mct1 expression in SHR astrocytes may reduce the intracellular availability of butyrate required to regulate expression of genes and maintain appropriate mitochondrial function within the brain, similar to what happens in certain gut diseases (Thibault et al. 2010). Surprisingly, treatment with butyrate decreased relative expression levels of Mct1 (Borthakur et al. 2008), contrary with previous reports in other tissues suggesting that presence of butyrate increased expression of Mct1, but only in the S‐D astrocytes, again highlighting the apparent lack of response of SHR astrocytes to butyrate application. Considering the role of astrocytes in maintenance of neuronal homeostasis, improvement in astrocyte mitochondrial function may be potentially beneficial to neurons in HTN (Hayakawa et al. 2016); however, butyrate‐based treatments for HTN may be hampered if there is dysfunctional expression of metabolite transporters, receptor signaling, or mitochondrial damage.

Inflammatory cytokines were also responsive to butyrate in astrocytes, and this was also dependent upon the rat strain. Various immunity‐related genes have been associated with butyrate regulation (Mathew et al. 2014; Dengler et al. 2015). Unsurprisingly, astrocytes cultured from SHR showed upregulated levels of Ccl2 and Tlr4 inflammatory markers, both associated with HTN in the SHR (Santisteban et al. 2015). Notably, and contrary with butyrate effect on mitochondrial function in the SHR, the expression of Ccl2 and Tlr4 markedly decreased following treatment with butyrate, but only in the SHR astrocytes. This suggests that central anti‐inflammatory effects of butyrate may be both mitochondria‐ and Mct1‐independent. It is possible that the anti‐inflammatory effects of butyrate in SHR astrocytes may be AT1 receptor‐mediated. Indeed, ANG II has a well‐established role in initiation of neuroinflammatory responses in HTN (Ando et al. 2004; Santisteban et al. 2015), and, as shown here, the levels of AT1 receptors also downregulate in the SHR astrocytes following butyrate treatment. Thus, this central anti‐inflammatory effect of butyrate may have a significant application in HTN therapy. The role of Ccl2 in chemoattraction of inflammatory cells and particularly monocytes has previously been reported (Deshmane et al. 2009). Considering that activated immune cells from the periphery are able to infiltrate brain cardioregulatory regions in HTN (Santisteban et al. 2015), this highlights a potential role for gut microbiota and its metabolites in either promoting or initiating central immune responses in HTN. Tlr4, also downregulated by butyrate in the SHR model, is involved in recognition of bacterial fragments such as LPS (Okun et al. 2014), and its role in neurogenic HTN is well documented (Dange et al. 2015). All told, our current data suggest that butyrate, lack of which has been proposed in the SHR (Yang et al. 2017), may have a beneficial effect on proinflammatory factors in the brain of SHR.

Interestingly, Olf59 is significantly overexpressed in the SHR astrocytes, and reduced with butyrate treatment in SHR astrocytes only. Considering the role of astrocytes and butyrate in blood brain barrier function (Abbott et al. 2006; Braniste et al. 2014), the overexpression of Olf59 may be an indication of lower butyrate availability in SHR circulation and the consequent compensatory upregulation of olfactory systems. Further studies are needed to investigate this possibility. Conversely, Tnf increased in expression with butyrate in astrocytes from SHR but did not the S‐D rats. Upregulation of Tnf has a reported role in HTN (Sriramula et al. 2013; Song et al. 2014; Hernanz et al. 2015), which made our results unforeseen. However, Tnf is a complex mediator involved in both beneficial and deleterious effects of the immune system (Barbara et al. 1996; Tian et al. 2014), and further studies are needed to fully elucidate the mechanisms of butyrate control of Tnf expression in the hypertensive brain. Lastly, there was no change in expression levels for Hmgcs1, Oxct1, Oxct2a, or Atct1 in astrocytes of either strain following treatment with butyrate, molecules involved in various forms of utilization of butyrate, suggesting that butyrate may not directly influence these molecules under the conditions examined.

Investigation into the therapeutic benefits of butyrate continues, as new evidence emerges on the role of this SFCA in maintenance of host homeostasis. Data presented here show that there can be significant differences in astrocyte metabolism between the two strains, with SHR showing evidence for dysfunction in response to butyrate treatments, in support of the hypothesis that astrocytes are under stress in this hypertensive rat model. The gut microbiome has been proposed to play a role in pathophysiology of HTN, and microbial metabolites produced in the gut can enter the blood, cross the blood brain barrier, and influence the nervous system. These metabolites can therefore be a significant regulator of cells in the CNS.

This study improves understanding as to the role of microbial metabolites in regulating astrocytes, and suggests that butyrate can regulate the bioenergetics of astrocytes as well as affect transcripts related to neuroinflammation. These mechanisms are proposed to underlie differences in astrocytes between normotensive and hypertensive animals. We did not directly compare baseline mitochondrial function between S‐D and SHR cultures, as each model was examined in a single plate in the assay and direct comparison of OCR across plates can be challenging. However, each plate was treated and run in a similar manner and time, and contained the same amount of cells. Our data do suggest differences in response to our butyrate treatments, making a case for inherent differences in mitochondrial function between the two strains. Future studies have been designed to confirm our current results in an *ex vivo* brain slice model obtained from adult normotensive and hypertensive rats.

One interesting question is whether the pups are exposed to any levels of butyrate before euthanasia, leading to the different levels between SD and SHR. As per our standard methods, the pups are 2 days old when euthanized, during which time they are not exposed to exogenous butyrate. However, it is plausible that the pups are exposed to butyrate and other SCFAs in utero, considering the possibility of inherent differences in production of SCFAs in the two strains used in this experiment. However, it has been suggested that, although fatty acids are able to cross the placenta during pregnancy, this affinity is reduced with SCFAs and increased with the number of double bonds (Gil‐Sanchez et al. 2011). It is also plausible that SCFAs are transferred to pups in mother's milk prior to weaning. Other studies have demonstrated the presence of SCFAs in bovine milk, for example, (Mansson 2008) but not in human milk (McNabney and Henagan 2017); we are not aware of any data on the presence/absence of SCFAs in rodent milk. This is an interesting question nevertheless that can be investigated in future experiments. A final point to make is in terms of gene expression, and butyrate is a nonspecific histone deacetylase inhibitor. This has implications for the global epigenetic regulation of genes by inhibiting a broad type of histone deacetylase (Dokmanovic et al. 2007). Therefore, the expression level changes in the genes here may be mediated through the epigenetic effect of butyrate, and this possibility requires further investigation.

## Conflict of Interest

The authors have no conflict of interest to declare.
